# The role of macrophages polarization in sepsis-induced acute lung injury

**DOI:** 10.3389/fimmu.2023.1209438

**Published:** 2023-08-24

**Authors:** Ziyi Wang, Zhong Wang

**Affiliations:** Beijing Tsinghua Changgung Hospital, School of Clinical Medicine, Tsinghua University, Beijing, China

**Keywords:** macrophages polarization, SALI (sepsis-induced acute lung injury), ARDS (acute respiratory distress), therapy, inflammation

## Abstract

Sepsis presents as a severe infectious disease frequently documented in clinical settings. Characterized by its systemic inflammatory response syndrome, sepsis has the potential to trigger multi-organ dysfunction and can escalate to becoming life-threatening. A common fallout from sepsis is acute lung injury (ALI), which often progresses to acute respiratory distress syndrome (ARDS). Macrophages, due to their significant role in the immune system, are receiving increased attention in clinical studies. Macrophage polarization is a process that hinges on an intricate regulatory network influenced by a myriad of signaling molecules, transcription factors, epigenetic modifications, and metabolic reprogramming. In this review, our primary focus is on the classically activated macrophages (M1-like) and alternatively activated macrophages (M2-like) as the two paramount phenotypes instrumental in sepsis’ host immune response. An imbalance between M1-like and M2-like macrophages can precipitate the onset and exacerbate the progression of sepsis. This review provides a comprehensive understanding of the interplay between macrophage polarization and sepsis-induced acute lung injury (SALI) and elaborates on the intervention strategy that centers around the crucial process of macrophage polarization.

## 1 Introduction

Sepsis, which has a high morbidity and mortality rate, results from infection caused by maladaptive immune and metabolic responses (1, 2). Dysregulation of the immune response leading to organ dysfunction and eventually organ failure is thought to be the key to distinguishing sepsis from infection and is increasingly recognized as an important part of the pathogenesis of sepsis (3–5). Early detection or intervention of Inflammatory response disorder may be a promising way to effectively attenuate sepsis (6–8).

Acute lung injury is a common complication of sepsis and a major cause of short-term death and long-term decline in quality of life (9). The pathogenesis of sepsis-induced acute lung injury (SALI) includes damage to the vascular endothelium and alveolar epithelium, leading to increased permeability of alveolar capillaries and decreased alveolar surface active substances (10, 11). According to relevant epidemiological studies, the incidence of ALI in sepsis patients is 68.2%, and the 90-day mortality rate of patients with combined ALI is as high as 35.5% (2).

**Figure 1:**
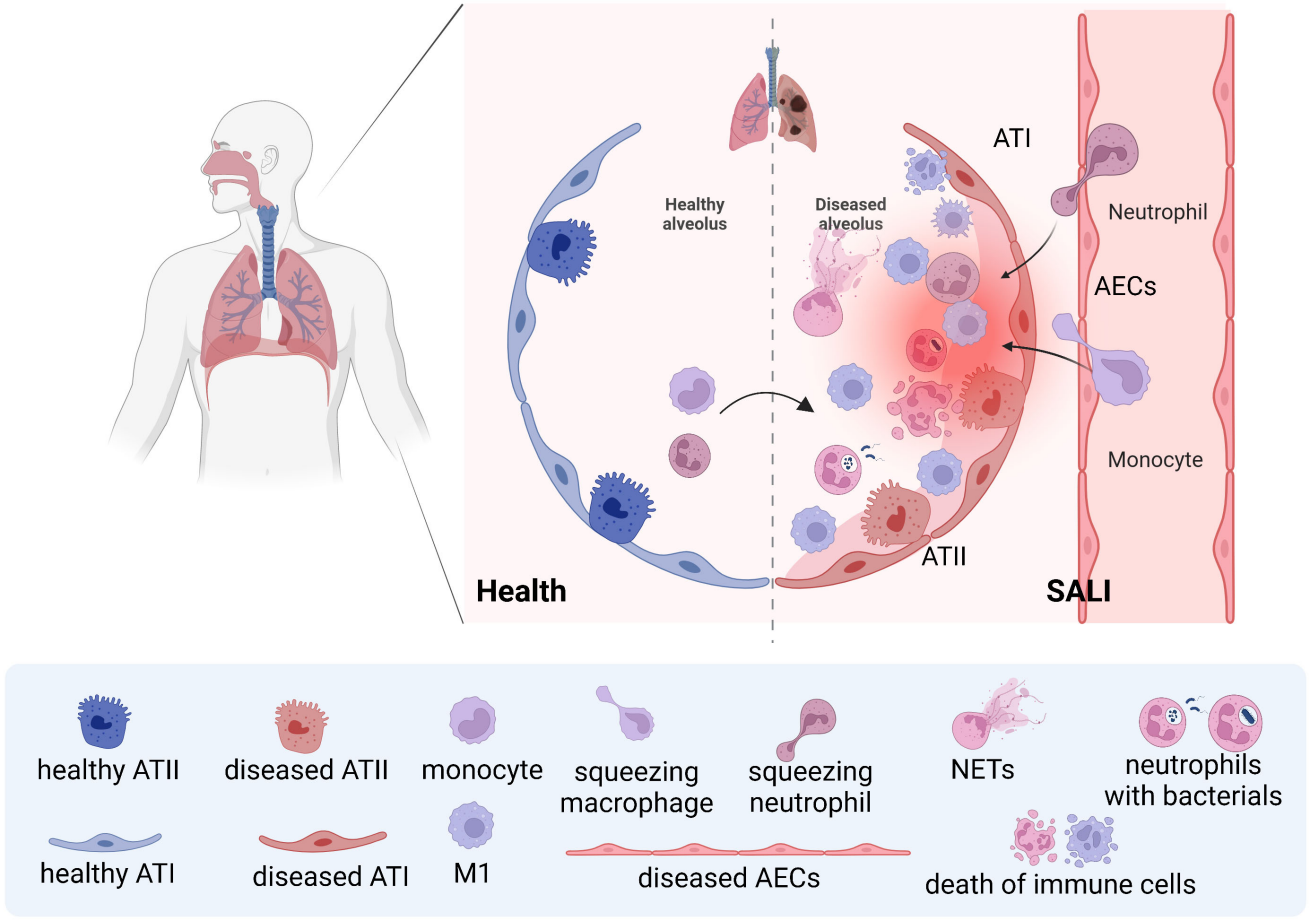
Schematic of neutrophils and macrophages being recruited during SALI During septic lung injury, neutrophils and monocytes are recruited from the blood into the lung, and monocyte-derived macrophages and resident macrophages work together to clear pathogens. However, the uncontrolled storm of inflammatory factors caused by effector cells will lead to further aggravation of SALI.

After invasion of lung tissue by endotoxins, microorganisms, and other pathogens, a large number of inflammatory factors are released, which activate effector cells such as alveolar macrophages, epithelial cells, endothelial cells, and multinucleated leukocytes, resulting in the release of an un-controlled inflammatory cascade. These can directly and indirectly damage alveolar epithelial cells and vascular endothelial cells, affecting gas exchange in the lungs, causing severe lung injury and reduced lung function (**Figure 1**) (12). Macrophages play dual roles in SALI. It is found that the total number of macrophages increased significantly after exposure to lipopolysaccharides (LPS), mainly increased inflammatory M1 macrophages and the corresponding M1 expression products, and decreased anti-inflammatory M2 macrophages and the corresponding M2 expression products (13).The mass production of M1-type macrophages will lead to the exacerbation of lung inflammation and alveolar damage, thus aggravating the degree of SALI. Otherwise, the production of M2-type macrophages can promote alveolar repair and regeneration, thus promoting SALI recovery. Therefore, in the treatment of SALI, the production and action of M1 and M2 type macrophages should be balanced to achieve the best therapeutic effect (14).

This article reviews the role of macrophage polarization in the immune response, summarizes the macrophage phenotype and the regulation of macrophage polarization, and explains the possible therapeutic approaches of targeting macrophage polarization in SALI. The purpose of this review is to comprehensively understand the function and characteristics of macrophage polarization in the immune mechanism of SALI. We will continue to review valuable targets and therapeutics related to the regulation of macrophage polarization and phenotypic changes.

## 2 Macrophage polarization

### 2.1 Monocytes-derived macrophages and native macrophages

**Figure 2:**
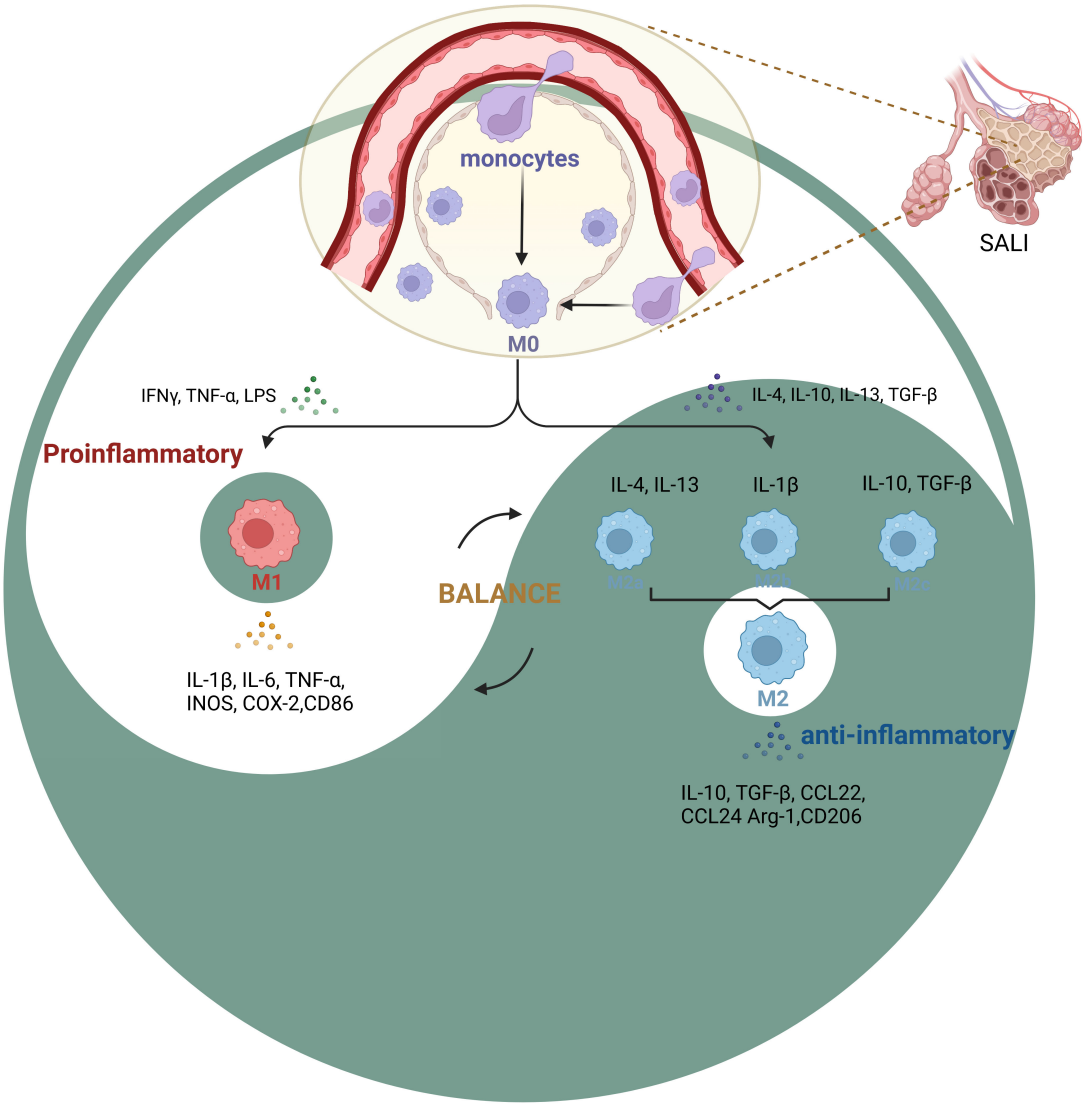
Phenotypic changes in macrophage polarization during SALI During inflammation, monocytes are recruited into the lung interstitium, bronchi and alveoli to be macrophages (M0), various tissue factors can induce their conversion into different phenotypes(M1, M2a, M2b, M2c et al.). The key to SALI therapy is the balance between M1 pro-inflammatory cells and M2 anti-inflammatory cells (Yin-Yang balance).

Activated macrophages are pathological markers of the immune and inflammatory responses of ALI (15, 16). According to recent studies, a considerable amount of tissue macrophages can be sustained independently of blood monocytes (17). This is in contrast to the previous assumption that macrophages originated solely from bone marrow hematopoietic stem cells. Instead, it is now understood that fetal liver monocytes, which are developed from late erythroid-myeloid progenitor cells produced in yolk sacs, also give rise to macrophages (18). There are four subtypes of pulmonary macrophages. These are alveolar macrophages (AMs), pulmonary interstitial macrophages (PIMs), pulmonary intravascular macrophages (PICMs), and dendritic cells (DCs). When activated, pulmonary macrophages can synthesize and secrete a large number of different substances. These secreted products are not only involved in protective reactions, but also related to tissue destruction (19). AMs account for about 95% of the white blood cells in alveolar cells, which originate during embryonic development and can self-renew throughout life (20). AMs is an important cell line in the catabolism of surface-active substances produced by alveolar type II epithelial cell (ATIIs) (21). As an important effector cell for lungs to resist foreign stimulation, AMs play a key role in the pathogenesis of lung inflammation (22). Interstitial macrophages (IMs) are situated in the connective tissue surrounding the bronchial airway (23). There are two sources of AMs. One is that monocytes in blood migrate into alveoli and differentiate into AMs, called monocytes-derived AMs. The second is derived from the division and proliferation of resident AMs. When inflammation occurs, the cell source is mainly the former. At present, it is believed that AMs are the main initiating cell of local inflammatory response in the lung. These recruited macrophages initially exhibit a different phenotype than resident macrophages. After activation, Various tissue factors can induce their conversion into macrophages with similar characteristics to resident macrophages (24). AMs produces a large number of inflammatory mediators, and can cause the activation of other immunocompetent cells and the release of inflammatory factors, leading to the uncontrolled local inflammatory response in the lung, which is an important cause of acute lung injury (25) (**Figure 2**).

### 2.2 Phenotype and function of macrophages

Macrophages can be categorized based on changes in the microenvironments of different tissues. However, the main phenotypes that are currently known are inflammatory or classically activated (M1-like) macrophages and healing or alternatively-activated (M2-like) macrophages (**Figure 2**). Upon exposure to external stimuli, they exhibit high antigen-presenting activity and pro-inflammatory phenotype, which helps eliminate infection-causing bacteria, fungi, and viruses. The M1-like macrophages are primarily involved in inflammation and immune response, which display high levels of interleukin (IL)-1β, IL-6, tumor necrosis-like factors (TNF)-α, and other inflammatory mediators along with inflammation-related genes such as inducible nitric oxide synthase (iNOS) and cyclooxygenase (COX)-2 (26, 27).

M2-like macrophages comprise three subpopulations: M2a, M2b, and M2c, which respond to different stimuli. M2a macrophages are induced by IL-4 and IL-13, while M2b macrophages are activated by IL-1β. M2c macrophages are stimulated by anti-inflammatory agents including glucocorticoids, IL-10, and transforming growth factor (TGF)-β. However, all three subpopulations express high levels of CD11 and anti-inflammatory factors such as IL-10, CCL24, and CCL22, which help prevent inflammation and pro-inflammatory immune responses (28). Also, M2-like macrophages mainly show high production levels of IL-10, TGF-β, and other anti-inflammatory factors, and also express high levels of arginine (Arg)-1, CD206, and other anti-inflammatory related genes (29). This effect inhibits tissue and cell inflammation and promotes tissue repair and angiogenesis (30).

AMs can carry out various functions depending on their ontology and receptor signaling in the microenvironment (31). Controlling how macrophages change their behavior could be a beneficial strategy for restoring the immune system balance in patients with sepsis, which could provide a fresh approach to treating various phases of the condition (32). Studies in recent years have found that macrophages can express a series of pattern recognition receptors including toll-like receptor (TLR), inflammatory body, and lectin-like receptors that are strategically located in the cell membrane, cytoplasm, and endometrium, and thus mediate the polarization of macrophages (33). M2 macrophages can inhibit the inflammatory response and play a role in tissue repair and reconstruction in the later stage of inflammation by secreting CXC chemokines ligand (CXCL)-12, IL-1α, tissue inhibitor of metalloproteinase (TIMP)-1, IL-4, and CXCL1 (34). The polarization process involves a variety of molecular mechanisms, mainly including TLR4/nuclear factor kappa B (NF-κB), janus kinase (JAK)/signal transducer and activator of transcription (STAT), TGF-β/Smads, peroxisome proliferator-activated receptor γ (PPARγ), Notch, and miRNA(miR) signal transduction pathways and inflammatory factors (35). By regulating the synthesis and release of various inflammatory mediators, macrophages seriously affect the development of ALI after infection and non-infectious stimulation.

## 3 The regulation of macrophage polarization in SALI

### 3.1 Regulation of macrophage polarization during the overwhelming inflammation phase of SALI

In the overwhelming inflammatory response phase of sepsis, targeted inhibition of M1-like macrophages can significantly reduce the release of inflammatory factors, thereby reducing tissue damage and patient mortality. The acute lung injury model is mostly induced by LPS or cecal ligation and puncture (CLP), and the overwhelming inflammatory response phase is mostly observed within 24 hours after treatment (**Table 1**).

**Table 1:** Pathways regulate macrophage polarization in different phases of SALI.

Phase of SALI	Pathway	Mode	Time to build model	Function	Reference
Overwhelming inflammation					
	MMP-9/NF-κB	CLP	24h	promote M1-like phenotype	(1)
	SENP3/HIF-1α/PKM2	LPS intraperitoneally	24h	promote M1-like phenotype	(2)
	Notch1	CLP	24h	inhibit M1-like phenotype	(3)
	ERK and NF-κB	CLP	24h	inhibit M1-like phenotype	(4)
	GADD34/IKKβ	LPS intraperitoneally	16h	inhibit M1-like phenotype	(5)
	MCP-induced Protein 1/JNK/c-Myc	CLP	6h	promote M2-like phenotype	(6)
	α-KG/PPARγ	LPS intraperitoneally	3h	inhibit M1-like phenotype	(7)
Immune suppression					
	eCIRP/IL-6R/STAT3	CLP	96h	promote M2-like phenotype	(8)
	miR-221 and miR-222/Brg1	LPS intraperitoneally	72h	promote M2-like phenotype	(9)
	Tim-3/IRF-7/IFN-α/β	LPS intraperitoneally CLP	24h	inhibit M2-like phenotype promote M1-like phenotype	(10)
	EPO/PI3K/AKT	LPS intraperitoneally twice	20h+6h	promote M2-like phenotype	(11)
	p21/NF-κB	LPS intraperitoneally twice	16h+2h	promote M2-like phenotype	(2)
	AMPK/TGF-β1	CLP	48–72 h	inhibit M2-like phenotype	(12)
	TLR4	LPS intraperitoneally for 5 consecutive days	5d	inhibit M2-like phenotype promote M1-like phenotype	(13)
Pulmonary fibrosis					
	EZH2/STAT/SOCS PPARγ	LPS intratracheally	3d/7d/14d	inhibit M2-like phenotype promote M1-like phenotype	(14)
	JNK	LPS intraperitoneally for 5 consecutive days	5d	promote M1-like phenotype	(15)
	IL-10 secreted by neutrophils	LPS intratracheally	32h	promote M2c-like phenotype	(16)

MMP-9, Matrix Metalloproteinase-9; NF-κB, nuclear factor kappa-B; CLP, cecal ligation and puncture; SENP3, SUMO1/sentrin/SMT3 specific peptidase 3; HIF-1α, Hypoxia-inducible factor 1α; PKM2, Recombinant Pyruvate kinase isozymes M2; Notch1, Notch homolog 1; ERK, extracellular regulated protein kinases; GADD34, Growth arrest and DNA damage-inducible 34; IKKβ, IKB kinase; MCP-induced Protein 1, Monocyte Chemoattractant Protein-1; JNK, c-Jun N-terminal kinase; c-Myc, cell-myc; STAT, signal transducer and activator of transcription; α-KG, D-alpha-Hydroxyglutaric acid disodium salt; PPARγ, peroxisome proliferator-activated receptor γ; STAT3, signal transducer and activator of transcription 3; eCIRP, extracellular cold inducible RNA binding protein; IFN, Interferon; AMPK, adenosine 5’-monophosphate -activated protein kinase; TGF-β1, transforming growth factor-β1; TLR4, Toll-likereceptor4; EZH2, enhancer of Zeste Homolog 2; SOCS, suppressor of cytokine signaling.

In SALI, the polarization of M1 macrophages is related to the activation of NF-κB and STAT1 signaling pathways (36). Tong et al. by studying mice 24 hours after CLP surgery, recently discovered that higher levels of MMP-9 in monocytes of early-stage sepsis patients correlate with the severity of the disease. Matrix Metalloproteinase-9 (MMP-9) promotes the polarization of macrophages to the M1-like phenotype by activating the NF-κB pathway. Furthermore, the Human immunodeficiency virus (HIV) protease inhibitor saquinavir can alleviate acute lung injury in septic mice by reducing the expression of MMP-9, thereby targeting the regulation of macrophage polarization (37). He et al. team concluded from observing the changes in the LPS-induced sepsis mice model 24 hours after LPS injection that Sentrin/SUMO-specific protease 3 (SENP3) promotes M1 macrophage polarization and the production of pro-inflammatory cytokines via the hypoxia-inducible factor 1α (HIF-1α)/Pyruvate kinase isozymes M2 (PKM2) axis, thereby contributing to lung injury; hence, SENP3 could represent a potential therapeutic target for LPS-induced ALI treatment (38). Zhou et al. found through studying mice 24 hours after CLP surgery that the compound 3,4-dihydroxyphenylethanol glycoside inhibits the polarization of M1-like macrophages by preventing the activation of the Notch1 signaling pathway, thereby significantly reducing the severity of acute lung injury in septic mice (39). Zhang et al. also studying mice 24 hours after CLP surgery, concluded that the extracellular regulated protein kinases (ERK) and NF-κB pathways promote M1 polarization, and loganin down-regulates the release of macrophage-associated M1 pro-inflammatory cytokines by inhibiting ERK and NF-κB pathways (40). In a separate study, Zhang et al. reported that the monocyte chemotactic protein (MCP)-induced Protein 1, a ribonuclease, can reduce sepsis-induced acute lung injury and mortality by targeting and regulating the polarization of M2-like macrophages via the c-Jun N-terminal kinase (JNK)/cell-myc (c-Myc) signaling pathway. This conclusion was drawn from observing mice 6 hours after CLP surgery (41). α-ketoglutarate (α-KG) downregulates the expression of m1 polarization marker genes and inhibits the activity of mammalian target of rapamycin complex 1 (mTORC1)/p70 ribosomal protein S6 kinase (p70S6K) signaling pathway in M1-like macrophages (42). Liu et al. illustrated that α-KG promotes il-4 induced M2 polarization of MH-S cells by increasing peroxisome PPARγ nuclear translocation and increasing the expression of fatty acid metabolism related genes, and reduces LPS-induced inflammation and lung pathological damage (42, 43).

### 3.2 Regulation of macrophage polarization in the immune tolerance phase of SALI

A subset of sepsis patients experiences a period of immunosuppression characterized by reduced output of inflammatory cytokines, increased secondary infections, and an increased risk of organ failure and death (44–47). During the immunosuppressive stage of sepsis, in addition to the apoptosis of a large number of immune cells, the polarization direction of macrophages also changed. M2-like macrophages secrete large amounts of anti-inflammatory mediators such as IL-10 and TGF-β, leading to host immune paralysis and severe infection recurrence. Therefore, targeted regulation that enhances M1-like macrophage polarization or reduces M2-like macrophage polarization may provide new therapeutic modalities for the immunosuppressive phase of sepsis (**Table 1**).

Zhou and others have found that extracellular cold inducible RNA binding protein (eCIRP) promotes endotoxin tolerance in macrophages. Using recombinant mouse CIRP (rmCIRP) to pretreat macrophages can demonstrate tolerance to LPS stimulation by reducing the production of TNF-α. The eCIRP promotes endotoxin tolerance and M2 polarization by activating p-STAT3 through IL-6R. Targeting eCIRP seems to be a potential treatment for correcting the immune tolerance of sepsis (48).

Specific microRNAs can regulate macrophage tolerance and may serve as biomarkers for immune paralysis and poor prognosis in sepsis patients (49). Long-term exposure to endotoxins can induce an innate immune memory, which weakens the subsequent response to unrelated pathogens, this is known as Endotoxin Tolerance (ET) (50). Peripheral macrophages may be a key factor in carrying peripheral antigens to the thymus medulla and affecting the negative selection of T cell populations to promote the formation of ET. These results suggest that clonal selection of ET in the thymus may provide protection against microbial sepsis (51). LPS tolerance encapsulates several key features of sepsis-associated immune suppression. Seeley and others screened tolerance-associated microRNAs and identified miR-221 and miR-222 as regulatory factors in the functional reprogramming of macrophages during the LPS tolerance process. Chronic stimulation of mice with LPS results in increased expression of miR-221 and miR-222, both of which regulate the Brahma related gene 1 (Brg1) (49).

Immune suppressive molecules regulate the functions of immune cells, decide the suppressive state of immune cells, and are key mediator molecules in the pathophysiology of sepsis (52). Tim-3 is also constitutively expressed on macrophages and dendritic cells and regulates the innate immune response (53, 54). In the late stage of sepsis, the expression of Tim-3 decreases, inhibiting M2 macrophage polarization and promoting M1 macrophage polarization, leading to a decrease in the release of anti-inflammatory agents and avoiding immune paralysis (55). Erythropoietin (EPO) is a glycoprotein regulated by HIF-1α and has anti-inflammatory and tissue protective functions. EPO improves host protective genes in endotoxin-tolerant macrophages and mice, such as antimicrobial peptide-related gene Cnlp, macrophage receptor with collagenous structure (Marco), and tissue repair gene vascular endothelial growth factor C (Vegfc), and this effect of EPO is regulated by PI3K-AKT (56). The authors found that p21 regulates macrophage reprogramming by altering the balance of the p65-p50 and p50-p50 NF-κB pathways. In human monocytes with p21 knocked out, M2-like macrophage polarization is weakened and the state of immune suppression in the body is reduced (57). Adenosine monophosphate-activated protein kinase (AMPK) participates in inhibiting the development of endotoxin tolerance, which is a driving factor for immune suppression induced by sepsis. AMPK activation inhibits LPS-induced TGF-β1 production and its signaling pathway, thereby inhibiting the development of endotoxin tolerance in macrophages (58). Liu et al. found that AMPK activation inhibited the development of LPS-induced endotoxin tolerance by repressing the accumulation of the immunosuppressive transcription factor hypoxia-inducible factor (HIF)-1α (59). AMPK activators have the potential to become therapeutic drugs for SALI. Natural compound resveratrol induces AMPK activation through the calcium/calmodulin dependent protein kinase kinase (CaMKK) pathway and inhibits the development of endotoxin tolerance by suppressing LPS-induced expression of IL-1 receptor-associated kinase M (IRAK-M) and the SH2-containing inositol-5-phosphatase 1 (SHIP1) (60).

Hydroxyapatite nanoparticles (HANPs) show moderate immunogenicity and can cause an innate immune response, which may involve the activation of Toll-like receptor 4 (TLR4) (61). In a mouse model of endotoxin tolerance, HANPs eliminate macrophage endotoxin tolerance by restoring the production of pro-inflammatory cytokines in macrophages in response to secondary LPS stimulation, and they enhance the body’s responsiveness to LPS re-challenge. HANPs can induce the activation of TLR4 signaling. In addition, HANPs dose-dependently cooperate with LPS to program LPS-induced macrophage TLR4 signal transduction, promote macrophage polarization to M1 phenotype, and eliminate macrophage immune tolerance to repeated LPS stimulation (62). This research provides a window into the intrinsic mechanism of HANPs driving the immune response.

### 3.3 Macrophage polarization in fibrotic phase of post-SALI

Pulmonary fibrosis is a late manifestation of acute respiratory distress syndrome (ARDS) (63). The morbidity and mortality of ARDS is especially high when it leads to persistent intra-alveolar and interstitial fibrosis (64, 65).

The model of pulmonary fibrosis after SALI is mostly induced by continuous administration of LPS through the trachea for 14 days. In idiopathic pulmonary fibrosis, the model is induced by bleomycin, and M2 phenotype of lung macrophages promotes tissue repair and is a pro-fibrotic phenotype (66). In the pulmonary fibrosis caused by the late stage of SALI, there is no clear conclusion on which phenotype of macrophages is the pro-fibrotic phenotype. Most scholars believe that pulmonary fibrosis after SALI is related to M1 macrophage polarization. Bao et al. found through *in vivo* and *in vitro* experiments that inhibiting EZH2 could inhibit the differentiation of M1 macrophages and promote the differentiation of M2 macrophages by inhibiting the STAT/suppressor of cytokine signaling (SOCS) pathway and activating PPAR-γ (67). Xu et al. demonstrated that the suppression of TNF-α release and PFKFB3 expression barred the occurrence of LPS-induced pulmonary fibrosis *in vivo*. To conclude, this research uncovered that the TNF-α secretion from LPS-triggered macrophages could incite fibroblast aerobic glycolysis and lactate production. This suggests that the interaction between inflammation and metabolism within lung macrophages and fibroblasts could assume a critical role in the process of LPS-induced pulmonary fibrosis (68). However, some scholars believe that pulmonary fibrosis after SALI is related to polarization of M2 type macrophages. Ye et al. found in the bronchoalveolar lavage fluid exosomes of mice with LPS-induced pulmonary fibrosis that IL-10 secreted by neutrophils may cause macrophages to polarize towards M2c, which may be an important mechanism of fibrosis after ALI (69). The interaction between lung macrophages and fibroblasts promotes the development of sepsis-induced pulmonary fibrosis. These evidences provide a new perspective on the mechanism of sepsis-induced pulmonary fibrosis, and may become potential therapeutic targets in the future.

## 4 Potential therapies for macrophage polarization in SALI

### 4.1 Protein-protein regulation of macrophage polarization

**Figure 3:**
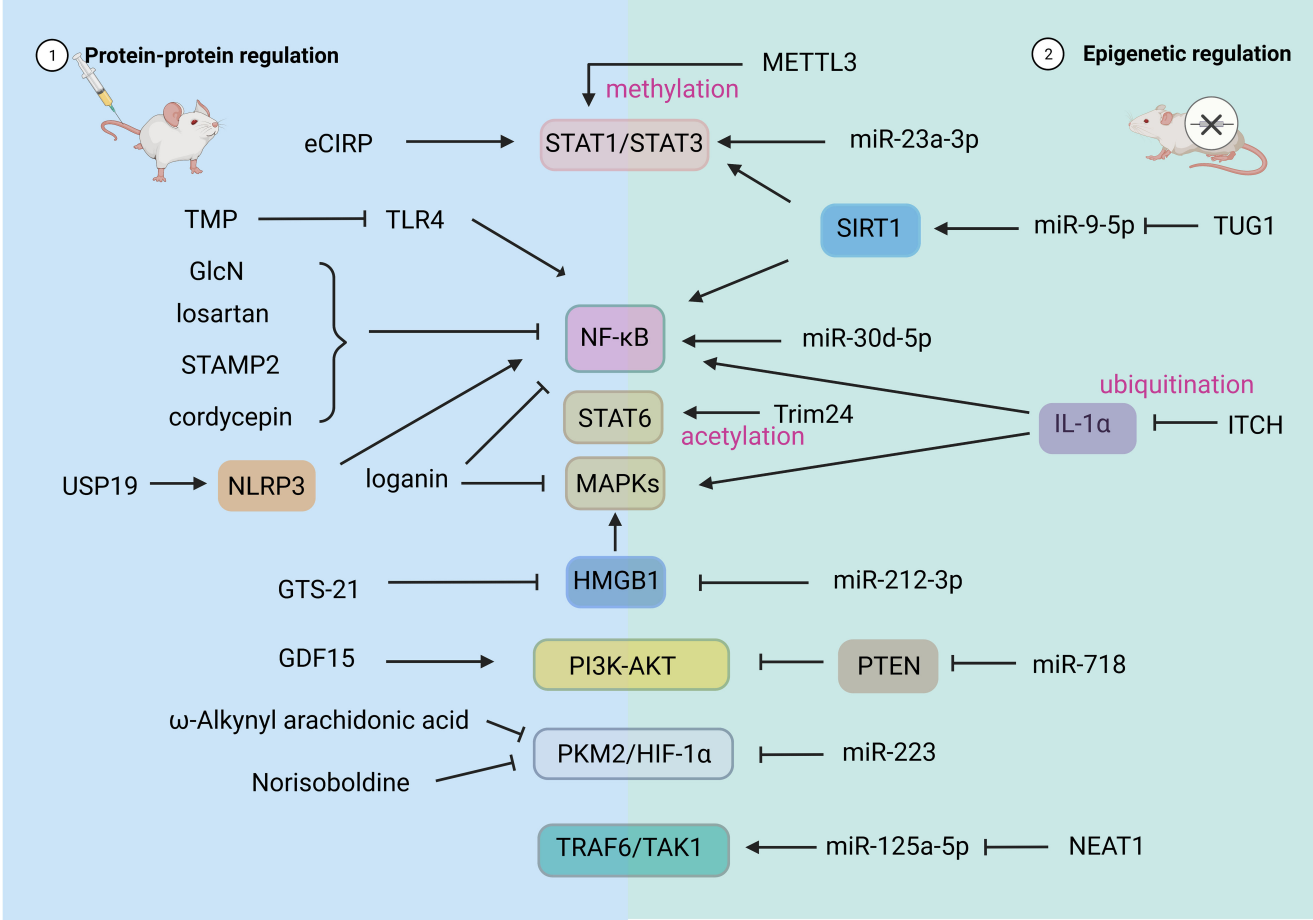
Drug and Epigenetic regulation of macrophage polarization. HIF-1α, hypoxia-inducible factor 1α; PKM2, recombinant pyruvate kinase isozymes M2; STAT, signal transducer and activator of transcription; AMPK, adenosine 5’-monophosphate -activated protein kinase; TRAF6, tumor necrosis factor receptor-associated factor 6; PTEN, phosphatase and tensin homolog deleted on chromosome ten; PI3K, phosphatidylinositol 3 kinase; AKT, protein kinase B, PKB. USP19, ubiquitin-specific protease; NLRP3, NOD-like receptor thermal protein domain associated protein 3. CBP, CREB-binding protein; METTL3, M6A-catalytic enzyme methyltransferase like 3; ITCH, E3 ubiquitin ligase.

In recent years, more and more studies have shown that related compounds, both artificial and natural, have significant effects on the regulation of the polarization state of macrophages in SALI (70). In the following, we review the related compounds and action pathways with high attention (**Figure 3**).

#### 4.1.1 Synthetic small-molecule compounds

As a new therapeutic strategy, small-molecule compounds are widely used in the treatment of SALI because of advantages such as simple molecular structure, high bioavailability, and low toxicity and side effects (71, 72). For instance, loganin effectively inhibits M1 macrophage polarization and NLRP3 inflammasome activation by blocking the ERK and NF-κB pathways (40). Prostatic 6 transmembrane protein 2 (STAMP2) may reduce the inflammatory response of SALI by inhibiting the activation of NF-κB signaling pathway and inhibiting M1 polarization of macrophages (73). In addition, cordycepin is crucial in mitigating lung injury in CLP mice by modulating the NF-κB signaling pathway to decrease the M1/M2 polarization of macrophages during sepsis. This underscores the significance of the NF-κB signaling pathway in ameliorating pro-inflammatory responses of macrophages (74). Cheng et al. showed that the synthetic analog of arachidonic acid, ω-alkynyl arachidonic acid, has the potential to induce M2 polarization of macrophages in acute myocardial infarction. This effect is achieved by regulating the interplay between PKM2, HIF-1α, and iNOS (75).

#### 4.1.2 Natural some-molecular compounds

Several natural compounds have been shown to promote the survival rate of mice and reduce damage to the alveolar structure and inflammation caused by SALI. These compounds work by improving the M1 phenotype of lung macrophages and promoting the M2 phenotype of lung macrophages. One example is quercetin, which enhances the polarization of M2 macrophages and boosts the expression of endogenous antioxidants in macrophages and microglia. It also lowers the levels of oxidative stress products such as NO, iNOS, and COX-2. Furthermore, it decreases the expression levels of M1 markers such as interleukin-6, tumor necrosis factor (TNF)-α, and IL-1β (76). Norisoboldine (an active ingredient in the root bark and bark of Lindera aggregate Kosterm) can attenuate SALI via promoting M2 polarization of macrophages through the PKM2/HIF-1α signaling pathway (77). The natural compound tetramethylpyrazine (TMP), an active ingredient extracted from ligusticum chuanxiong, alleviates ALI by blocking various signaling pathways including TLR4/TRAF6/NF-κB/NLRP3/caspase-1 and TLR4/caspase-8/caspase-3. This results in a reversal of macrophage polarization, a decreased in cell pyroptosis, and prevention of apoptosis (78).

#### 4.1.3 Other promising related compounds

Macrophage polarization is closely related to inflammatory response (79). Therefore, anti-inflammatory drugs may atteunate SALI by regulating macrophage polarization. Compared with M2-polarized AMs, M1-polarized AMs increased pulmonary inflammation (80). For instance, Tong et al. showed that saquinavir (SQV), a first-generation protease inhibitor used to treat HIV, has anti-inflammatory properties. Specifically, SQV was found to induce the M2 phenotype of lung macrophages in mice that had undergone cecal ligation and puncture (CLP) surgery. This effect was attributed in part to the inhibition of matrix metalloproteinase-9 (MMP-9), which helped to maintain a balance between M2 and M1 macrophage phenotypes (37). Gainesville–Tokushima Scientists (GTS)-21 has a protective effect against LPS-induced lung injury by reducing the expression level of HMGB1 in AMs, reducing the number of AMs, and reducing the level of AM-related inflammatory factors (81). Glucosamine (GlcN) inhibits LPS-induced O-GlcNAc expression in mouse lung and zebrafish visceral tissues to up-regulate proinflammatory cytokines through inhibition of NF-κB (82). Liu et al. demonstrated that ubiquitin-specific protease (USP19) switches the proinflammatory function of NOD-like receptor thermal protein domain associated protein 3 (NLRP3) into an anti-inflammatory function by promoting M2-like macrophage polarization through direct association with interferon regulatory factor 4 (83). Similarly, it has been reported that losartan can increase the M1 subtype of lung macrophages via the same signaling pathways. This helps to keep the balance of mitochondria in cardiomyocytes, lower oxidative stress, and reduce apoptosis of myocardial cells (84). Growth differentiation factor-15 (GDF15) with resistance to apoptosis, anti-inflammatory and endothelial protective effect. GDF15 regulates the polarization of macrophages to M2 by activating the PI3K/Akt signaling pathway, reduces the level of M1 in macrophages, and has a protective effect on the survival rate of sepsis mice model. The increase of serum GDF15 level is closely related to the severity and mortality of sepsis patients, and GDF15 can be used as a prognostic indicator of sepsis (85).

### 4.2 Epigenetic regulation of macrophage polarization

As shown in **Figure 3**, the regulation of non-coding RNA can affect the polarization state and function of macrophages by changing its gene expression (70). Jiao et al. reported that PMNs exosome miRNA (miR)-30d-5p induced polarization of M1 macrophages and pyroptosis of macrophages by activating the NF-κB signaling pathway, thus participating in the occurrence of SALI. These findings suggest a new mechanism of PMN-Mφ interaction in SALI and provide new therapeutic strategies for sepsis patients (86). Jiang et al. found that miR-23a-3p promoted the activation of STAT1/STAT3 by down-regulating Polo-like kinase 1 (PLK1) and increased the M1 polarization of macrophages. Inhibition of miR-23a-3p resulted in decreased macrophage response and promoted inflammation in ALI (87). miR-718 can directly down-regulate phosphatase and tensin homolog (PTEN) to activate PI3K/Akt phosphorylation, reduce M1 type transformation, resulting in reduced production of proinflammatory cytokines (88). miR212-3p was shown to directly target HMGB1 and further inhibits MAPK pathway, thereby inhibiting the LPS-induced inflammatory response of RAW264.7 cells (89). N6 - methyl adenosine (m6A) methylation is the most abundant mammalian mRNA epigenetic modifications. M6A-catalytic enzyme methyltransferase like 3 (METTL3) drives M1 macrophage polarization by directly methylating STAT1 mRNA and may serve as an anti-inflammatory therapeutic target (90). Ubiquitination is a post-translational modification that regulates these inflammatory signaling pathways. E3 ubiquitin ligase (ITCH) is a negative regulator of inflammation. Its reduction leads to ubiquitination of IL-1α which in turn leads to increased pro-inflammatory polarization of macrophages (91).

Regulation of polarization-related non-coding RNA in M2-type macrophages can promote the production and action of M2-type macrophages, thereby promoting alveolar repair and regeneration. During sepsis, the miR-223 level positively correlates with the proportion of M2 macrophages, and the clinical score of sepsis and mortality of miR-223 knockout mice increased in one study (92). In another study, the overexpression of miR-223 reduced the expression of iNOS in LPS-stimulated macrophages, decreased the expression of IL-6, and increased the expression of IL-10. Additionally, miR-223 interferes with the glycolysis pathway and promotes the expression of M2-type macrophages by down-regulating HIF-1α (93). Knockdown of lncRNA nuclear enrichment transcript 1 (NEAT1) promotes M2-type polarization of macrophages through the miR-125a-5p/TRAF6/TAK1 axis, thereby improving the LPS-induced inflammatory response (94). One study showed that extracellular vesicles (EVs) derived from endothelial progenitor cells (EPCs) promote the polarization of M2-type macrophages by lncRNA taurine up-regulated gene 1 (TUG1) through the destruction of miR-9-5p–dependent Sirtuin 1 (SIRT1) inhibition (95). STAT6 is known to drive macrophage M2 polarization. Yu et al. found that lysine (Lys) 383 of STAT6 is acetylated by the acetyltransferase CREB-binding protein (CBP) to suppress macrophage M2 polarization to contribute to the induction of an immunosuppressive tumor niche (83).

### 4.3 Metabolic reprogramming of macrophage polarization

Metabolic reprogramming encapsulates the alterations in a cell’s energy requirements. This is carried out to equip the cells with enhanced resilience against external environmental stressors and impart new functions to them. Such reprogramming incorporates changes in metabolism-associated products, enzymes, and metabolic pathways (96). The metabolic profile of M1 macrophages is defined by aerobic glycolysis, fatty acid synthesis, and a truncated variant of the tricarboxylic acid (TCA) cycle. Conversely, M2 macrophages exhibit characterization through fatty acid oxidation (FAO) and an oxidized version of the TCA cycle (97).

Research shows that the activation of macrophages by interferon (IFN)-γ and LPS results in the truncation of the TCA cycle at the levels of isocitrate dehydrogenase (IDH) and succinate dehydrogenase (SDH), leading to the accumulation of succinate and citrate metabolites. The accumulation of succinate in M1-like macrophages can stabilize HIF-1α by inhibiting the activity of proline hydroxylase (PHD), thereby promoting glycolytic metabolism and driving the inflammatory response (98, 99). LPS-induced succinate stabilizes hypoxia-inducible factor-1α, an effect inhibited by 2-deoxy-D-glucose, with IL-1β being an important target (98).

Accumulated citrate serves as the precursor of the macrophage-specific metabolite itaconate, which characterizes IFN-γ/LPS-polarized macrophages (100). Studies on mouse and human macrophages indicate that itaconate is the first example of a metabolite linked to the antibacterial function of pro-inflammatory macrophages (101). When itaconate generation is impaired, LPS-induced immune responsive gene 1 (IRG1) −/− mouse bone marrow-derived macrophages do not display succinate accumulation, further proving that itaconate is a specific driver for the accumulation of succinate in lipopolysaccharide-stimulated macrophages (102, 103). Studies also propose that itaconate produces therapeutic effects on SALI by inhibiting ferroptosis (104).

In macrophages, the main source of ROS is NADPH oxidase, which produces two superoxide radicals for each molecule of NADPH consumed (105). Scholars have pointed out that ROS is closely related to the pro-inflammatory phenotype M1 conversion of macrophages (106). During sepsis, FAM96A may mediate an immunometabolic shift in macrophages from oxidative phosphorylation (OXPHOS) to glycolysis, associated with reactive oxygen species (ROS) and glucose uptake (107). The inflammatory phenotype of these macrophages can be inhibited by blocking ROS production through uncoupling mitochondrial respiration with triptolide or by expressing alternative oxidase (AOX), which protects mice from LPS-induced lethality (98). However, studies have also indicated that the Th2-like cytokine IL-25 can induce ROS production, increase mitochondrial respiratory chain complex activity, subsequently activate AMPK, and induce M2 macrophage polarization in monocytes (108).

Arg can affect the polarization state of macrophages through two different metabolic pathways: the NO synthesis pathway and the Arg-1 pathway. Glutamine can widely enter the TCA cycle and the hexosamine pathway, promoting the polarization of M2-like macrophages under IL-4 stimulation (109, 110). In summary, the metabolic reprogramming of macrophages is a complex process. Elucidating the metabolic reprogramming of macrophages in an inflammatory environment would help to identify targets for the regulation of macrophage polarization in inflammatory diseases.

### 4.4 Regulation of macrophage polarization by mesenchymal stem cells

Mesenchymal stem cells (MSCs) have the ability to secrete substances, regulate the immune system, and promote tissue repair (111). These cells hold potential for treating both acute and chronic inflammatory lung diseases (112, 113). MSCs function by interacting with specialized immune cells called resident alveolar and interstitial macrophages (114). MSCs not only enhance the growth of undifferentiated M2 and pre-M2 by releasing macrophage colony-stimulating factor but also encourage M2 polarization of pre-M2 through direct contact with them (115). The microenvironment within the lungs encourages MSCs to release certain substances that lead to the transformation of macrophages into an M2-like phenotype that suppresses the immune system. This change supports the preservation of a steady and well-functioning tissue environment (116). A potential therapeutic strategy for sepsis was presented by Liu et al., where they showed that MSCs can produce TGF-β, which has the potential to shift the polarization of LPS-stimulated macrophages towards an M2-like phenotype. This shift leads to a decrease in inflammatory response and an improvement in phagocytosis, which is achieved through the AKT/foxo1 pathway (117). For example, Bai et al. found that extracellular vesicles from adipose tissue-derived stem cells can selectively inhibit M1-like macrophage polarization through the Notch-miR148a-3p signal axis, thus alleviating sepsis-induced inflammation and protecting organ function (118). Bone marrow mesenchymal stem cells (BMSCs) can alleviate sepsis-induced lung injury by secreting exosomes, and have powerful immunomodulatory and immunosuppressive properties. Deng et al. found that exosomes released by BMSCs administered through the trachea could inhibit M1-like polarization and promote M2-like polarization of MH-S cells (a mouse alveolar macrophage cell line), thus treating SALI (119).

## 5 Conclusion

This article reviews the phenotype of macrophage polarization and its regulation, and the role of macrophage polarization in SALI. Further understanding of the function and characteristics of macrophage polarization and its role in the host immune response in SALI may contribute to the development of new therapies targeting the different stages of SALI, as well as the regulation of immune homeostasis in the microenvironment under sepsis conditions.

During sepsis, the lung is highly susceptible to damage caused by inflammation, which is also one of the most easily affected organs by inflammatory factors. SALI is a condition where the lungs become inflamed and damaged because of various factors. Despite considerable advancements in early detection and organ-function assistance for patients with sepsis, the occurrence and fatality rates of sepsis remain elevated, presumably because of the challenging nature of reversing organ damage (16, 120–122). Therefore, early recognition and intervention of SALI are key factors in improving the prognosis of sepsis patients (123, 124). We can see that the role of small molecule compounds, including synthetic small molecule compounds and natural small molecule compounds in the prevention and blocking of sepsis has received increasing attention.

Tremendous evidence has shown that the study of macrophage polarization has a broad prospect, and its intervention strategy is also expected to be a new approach for the treatment of SALI. When exposed to changes in the microenvironment of different tissues, macrophages will express M1-like or M2-like phenotypes with different functions. The former can secrete large amounts of pro-inflammatory mediators, and while they provide defense against pathogens, they may also damage tissue or immune cells. In contrast, M2-like macrophages secrete a large amount of anti-inflammatory mediators, which can reduce the host inflammatory response and induce immune paralysis. Therefore, macrophage polarization regulation is particularly important for maintaining host immune homeostasis. However, due to the complexity of the macrophage polarization regulatory network, it is important to identify the signaling pathway and key transcription factors for the targeted regulation of this process. In addition, targeted regulation of macrophage polarization at the epigenetic level opens up a series of new therapeutic avenues for sepsis and related diseases. In clinical practice, appropriate intervention measures can be selected according to the specific conditions of patients to achieve optimum therapeutic effect. It needs to be pointed out in particular that, although the M1/M2 paradigm has provided a useful framework for understanding macrophage biology, macrophage activation exists on a diverse spectrum and cannot be easily defined by such phenotypic dichotomy, particularly *in vivo* where numerous stimuli may be present together.

At present, various approaches have been demonstrated to interfere with the occurrence and development of SALI by influencing the polarization state of macrophages. However, the precise regulatory mechanisms of macrophage polarization remain incompletely understood. Future research directions include: 1) searching for more small-molecule compounds that can regulate the polarization or apoptosis of macrophages; 2) exploration of biomarkers related to macrophage polarization that can identify progression to sepsis in the early stages of infection; 3) clinical trials to verify the therapeutic effect of new agents in SALI and 4) individualized precision treatment targeted towards different stages of pathogenesis and sepsis of SALI patients and combined with patients’ age, genetics, and underlying diseases should be carried out. Consequently, targeted regulation of macrophage polarization through protein-protein intervention, epigenetic modification, and metabolic reprogramming is expected to develop new approaches for the treatment of different stages of SALI.

## Author contributions

ZYW and ZW contributed to conception and design of the review. ZYW wrote and revised the manuscript. All the authors contributed to manuscript revision, read, and approved the submitted version.
